# An in-situ calibration method and the effects on stimulus frequency otoacoustic emissions

**DOI:** 10.1186/1475-925X-13-95

**Published:** 2014-07-08

**Authors:** Shixiong Chen, Haoshi Zhang, Lan Wang, Guanglin Li

**Affiliations:** 1 Institute of Biomedical and Health Engineering, Shenzhen Institutes of Advanced Technology, Chinese Academy of Sciences (CAS), Shenzhen, Guangdong 518055, China; 2 Key Laboratory of Human-Machine-Intelligence Synergic System, Shenzhen Institutes of Advanced Technology, Chinese Academy of Sciences (CAS), Shenzhen, Guangdong 518055, China

**Keywords:** Standing wave, Stimulus frequency otoacoustic emissions, Swept tones, In-situ calibration, Hearing loss

## Abstract

**Background:**

The interference between the incoming sound wave and the acoustic energy reflected by the tympanic membrane (TM) forms a standing wave in human ear canals. The existence of standing waves causes various problems when measuring otoacoustic emissions (OAEs) that are soft sounds closely related with the functional status of the inner ear. The purpose of this study was to propose an in-situ calibration method to overcome the standing-wave problem and to improve the accuracy of OAE measurements.

**Methods:**

In this study, the sound pressure level (SPL) at the TM was indirectly estimated by measuring the SPL at the entrance of the ear canal and the acoustic characteristics of the earphone system, so that sound energy entering the middle ear could be controlled more precisely. Then an in-situ calibration method based on the estimated TM SPL was proposed to control the stimulus level when measuring the stimulus frequency otoacoustic emissions (SFOAEs) evoked by swept tones. The results of swept-tone SFOAEs with the in-situ calibration were compared with two other calibration methods currently used in the clinic.

**Results:**

Our results showed that the estimate of the SPL at the TM was rather successful with the maximal error less than 3.2 dB across all the six subjects. With the high definition OAE spectra achieved by using swept tones, it was found that the calibration methods currently used in the clinic might over-compensate the sound energy delivered to the middle ear around standing-wave frequencies and the SFOAE amplitude could be elevated by more than 7 dB as a consequence. In contrast, the in-situ calibration did not suffer from the standing-wave problem and the results could reflect the functional status of the inner ear more truthfully.

**Conclusions:**

This study suggests that calibration methods currently used in the clinic may produce unreliable results. The in-situ calibration based on the estimated TM SPL could avoid the standing-wave problem and might be incorporated into clinical OAE measurements for more accurate hearing loss screenings.

## Background

The human ear canal is a tube with one end open to the air and the other end terminated by the tympanic membrane (TM) that separates the outer ear from the middle ear. When an incoming sound travels into the ear canal, part of the acoustic energy will be reflected by the TM and travels backward in an opposite direction as the forward waveform. The forward and backward waveforms can enhance each other if they are in phase, and cancel each other if they are out of phase [1-3]. The enhancements or cancellations between the forward and backward waveforms can form a standing wave (or stationary wave) in the ear canal, characterized by positions where the sound pressure level (SPL) appears to be standing still.

The existence of standing waves has large impacts on the quantification of the sound pressure in the ear canal. One significant impact would be that the actual SPL measured at the ear canal could be dramatically different from the stimulus levels generated from the computer [2]. On the other hand, the sound pressures measured at different locations along the ear canal may vary significantly, for example, the SPL measured at the entrance of the ear canal can be totally different from the SPL near the TM [4]. As a consequence, the standing wave would cause various problems and difficulties in the calibrations of stimulus levels in clinical audiologic measurements. Without taking into account the effects of standing waves, the hearing thresholds of standard audiogram tests may be questionable [5,6], the acoustic measurements of hearing aid fittings could cause over amplification and discomfort of the patients [7,8], and the results of otoacoustic emissions could be unreliable [9-12].Therefore, the actual sound energy delivered to the middle ear should be measured to quantify the stimulus level presented to the ear in clinical applications. A commonly accepted reference of the delivered sound energy is the TM pressure measured within a few millimeters from the TM [1,11].

Various calibration methods have been proposed to calibrate the stimulus level in audiologic measurements to solve the standing-wave problem. The calibration is a process during which the stimulus level is continuously adjusted until the measured SPL at a specific location achieved the desired level. If the stimulus is a wideband signal in hearing assessments, a flat spectrum is usually desired so that the results from different frequencies could be compared to identify the abnormal frequency range with possible hearing loss. One direct calibration method is to insert a tiny microphone within 2 mm of the TM to measure the actual TM sound pressure [1,11]. However, inserting a microphone so close to the TM could cause discomfort of the patients and might lead to potential threat of damaging the TM. This issue would be especially more difficult and vulnerable when working with a child. Another calibration method, called the probe calibration, is to set the stimulus level according to the SPL measured at the entrance of the ear canal. However, the SPL at the ear-canal entrance could be totally different from the desired SPL near the TM and the stimulus level could be over compensated by as much as 15 to 20 dB [11]. Whitehead *et al*. [13] used a “no calibration” strategy, in which the driving voltage of the earphone is set to be constant at the desired level without measuring the actual ear-canal SPL. However, there is no guarantee that sound energy delivered to the middle ear is as expected due to the impacts of the standing wave. Recently, a new type of calibration method called the forward pressure method has been developed and is under intense investigations [6,12,14]. The forward pressure method tries to separate the mixed waveform in the ear canal into two separate components: the forward pressure travelling along the ear canal to the middle ear and backward pressure reflected by the TM. Unlike the mixed ear-canal SPLs, the individual component of the forward pressure is theoretically free from the impacts of standing waves and it is therefore frequently used as the reference to quantify the sound energy transmitted to the middle ear [9,15,16]. However, there is no way to actually measure the forward pressure component anywhere in the ear canal and therefore the validity of the forward pressure method remains unclear.

Otoacoustic emissions (OAEs) are low-level sound energy produced by the outer hair cells in the cochlea, either spontaneously or evoked by external stimuli [17]. Since OAEs are easy to measure and the results can truthfully reflect the healthiness of the cochlea, the measurement of OAEs is widely used as a routine hearing screening tool in the clinic. Due to the impacts of the standing wave, it is necessary to calibrate the stimulus to ensure that the SPL actually measured in the ear canal achieved a desired target in OAE measurements. The effects of different calibrations on OAE measurements have been intensely investigated in many studies [9-12,15]. Some studies reported significant differences in OAE amplitudes among different calibration methods [10-12]. Siegel [11] reported that the calibration differences were more evident at high frequencies, and Whitehead [10] suggested that the effects were level dependent. However, other studies [9] found little or no significant effects on their OAE results across frequencies at any clinically applicable stimulus levels. A possible reason for these inconsistent findings is that all these studies used pure tones as the stimulus primaries, and the frequency range where OAEs demonstrate significant differences may not be covered by the insufficient number of discrete frequencies. Moreover, DPOAEs are considered to be a summation of two distinctive sources from two remote cochlear regions [18,19] and the complex generation mechanisms make the results of DPOAEs vulnerable to the interactions of the two sources. Therefore, another type of OAEs, the stimulus frequency otoacoustic emissions (SFOAEs), attracted intense attentions since they are sensitive to cochlear damages [20,21] and their generation mechanisms are simpler than DPOAEs [22,23], making them a great alternative tool for hearing loss screenings. Recently, Chen *et al.*[24] used a swept tone whose frequency changes continuously over time to measure the SFOAEs. It showed that the swept-tone method was capable of measuring numerous frequencies across a wide frequency range within a short period of time. The use of swept tones might make it probable to catch any slight OAE differences caused by different calibration methods, if any. According to our knowledge, there are no previous studies to investigate the effects of calibrations on SFOAE measurements by using swept tones.

The purpose of this study is to propose an in-situ calibration method to overcome the standing-wave problem and to examine its benefits in SFOAE measurements when compared with other calibration methods currently used in the clinic. The in-situ calibration was based on the TM SPL that was estimated by measuring the SPL at the ear-canal entrance and the acoustic characteristics of the earphone system. The proposed method could be useful to control the stimulus level more precisely and to improve the accuracy of the results in OAE measurements.

## Methods

### Estimate of sound pressure level at TM

#### Subjects

Six subjects (3 males and 3 females) with ages ranging from 20 to 32 years old (mean age = 25) were recruited in the experiments of the study. Each subject was screened with a custom OAE measurement program and the SFOAE amplitude was at least 5 dB above the noise level from 0.5 to 10 kHz (stimulus levels set at 65 dB SPL). All the subjects had normal hearing with behavior thresholds of 20 dB hearing level or less at standard frequencies from 250 Hz to 8 k Hz. The subjects were seated in a sound-proofed booth comfortably and told to be as quiet as possible during the tests. The recruiting and experimental protocols were approved by the Ethics Committee for Human Research of the Shenzhen Institutes of Advanced Technology, Chinese Academy of Sciences.

#### Equipment

**Figure 1:**
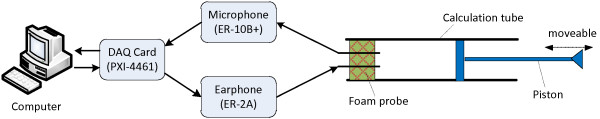
**Configuration of acoustic system.** The configuration of the acoustic system for the TM SPL eastimate and OAE measurements.

The configuration of the sound delivery and recording system was shown in Figure 1. A custom software program developed in Labview (National Instruments) was used to generate the stimuli from a personal computer (PC). The generated digital signal was converted to analog voltage by a 24-bit data acquisition and generation card PXI-4461 (National Instruments) to drive an ER-2A earphone (Etymotic Research) that had rather flat frequency response from 0.2 to 16 kHz. The acoustic response to the output of the earphone was recorded by a low-noise ER-10B + microphone (Etymotic Research) and then digitized by the PXI-4461 card at a sample rate of 48 k sample/s. The earphone and microphone was coupled inside an appropriately selected foam probe. A calculation tube was also used for the estimate of the SPL at the TM position. The calculation tube was a uniform plastic tube with one end open and the other end terminated by a rubber piston movable inside the tube (Figure 1). The tube was 7 mm in diameter (about the same size as the diameter of a human ear canal) and 150 mm in total length.

#### Stimulus

A swept tone constructed in the frequency domain [25] was used as the stimulus in this study. The swept tone was similar to the chirp signal (a wide-band signal with time-varying frequency) but its spectral contents could be freely customized, which was a helpful feature preferred by the stimulus calibrations in audiologic measurements. The frequency of the swept tone was increased linearly from 0.5 to 10 kHz within 1 s across all the experiments of this study. The swept tone was digitally generated from the computer and delivered to the ER-2A earphone to play the sounds.

#### Procedures

The general idea of the proposed method was that the SPL at the TM could be indirectly determined by the SPL at the entrance of the ear canal and the acoustic characteristics of the earphone system. The former could be easily measured by the ER-10B + microphone placed at the entrance of the ear canal (the same as conventional audiologic measurements), and the latter could be calculated by measuring several acoustic responses in a set of known acoustic loads [26].

**Figure 2:**
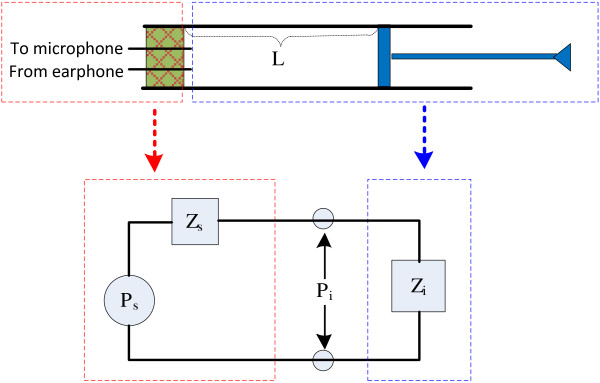
**Equivalent circuit of acoustic system.** The equivalent circuit of the transmission line of the acoustic system.

The calculation of the acoustic characteristics of the earphone system was crucial in estimating the SPL at the TM. It was achieved by modeling the transmission line of the acoustic system in Figure 1 as an equivalent circuit in Figure 2. In Figure 2, the earphone was considered as a power source with two parameters (source pressure *P*_*s*_ and source impedance *Z*_*s*_, both as functions of frequency) and the calculation tube was represented as a known acoustic load with an impedance of *Z*_*i*_[26]. The wideband SPL *P*_*i*_ at the load in response to a swept-tone stimulus (with a constant driving voltage of 0.2 V across frequencies from 0.5 to 10 kHz) was measured at the foam probe by the microphone. The variables in the equivalent circuit could be related by the following equation:

(1) $$\frac{P_{s}}{Z_{s}+Z_{i}}=\frac{P_{i}}{Z_{i}}$$

For a uniform tube with a known length, the acoustic impedance *Z*_*i*_ could be obtained by [27]:

(2) $$Z_{i}=-jZ_{0}\cot\left(2\mathrm{\pi fL}/c\right)$$

where $j=\sqrt{-1}$, *Z*_0_ = *ρc*(*ρ*: air density, *c*: sound speed in the air), and *L* is the effective tube length from the foam probe to the plate of the piston (see Figure 2). According to Equation (2), changing the effective tube length *L* could produce different known impedances *Z*_*i*_ and sound pressures *P*_*i*_, and in turn different sets of Equation (1). Therefore, the two unknown variables (*P*_*s*_ and *Z*_*s*_) could be solved by using two different *L*’s, achieved by moving the piston to two different positions. In practices, five or more different *L*’s were usually employed for more accurate results, by using a least square error method [12,14,26]. In this study, the piston of the calculation tube was gradually moved from 10 to 126 mm away from the foam probe at a step of 4 mm (Figure 2), resulting in 30 different effective tube lengths and correspondingly 30 different Equation (1). Then the 30 equations were randomly divided into 6 different groups, with each group consist of 5 equations to solve for *P*_*s*_ and *Z*_*s*_. Then the results from the 6 different solutions were compared to examine the reliability of the proposed method and the average of the solutions was used for subsequent calculations of estimating the SPL at the TM.

After the two parameters (*P*_*s*_ and *Z*_*s*_) of the earphone were solved by using the calculation tube, the foam probe was then inserted into the ear canal of each individual subject. The same swept-tone stimulus was presented and the SPL at the entrance of the ear canal was measured as *P*_*L*_. According to Equation (1), the impedance of the ear canal *Z*_*L*_ could be obtained by:

(3) $$Z_{L}=\frac{Z_{s}P_{L}}{P_{s}-P_{L}}$$

With the ear-canal impedance *Z*_*L*_ obtained, the SPL measured at the ear canal could be isolated into two components: the forward pressure (*P*_+_) that travels along the ear canal to the middle ear and the backward pressure (*P*_−_) that is reflected by the TM. The isolation of the ear-canal SPL made it possible to eliminate the standing-wave problem since the interactions between the two components were avoided. According to Scheperle *et al.*[12], the forward pressure *P*_+_ and backward pressure *P*_−_ of the SPL at the entrance of the ear canal (*P*_*L*_) could be obtained by:

(4) $$\left\{\begin{array}{c} P_{+}=\frac{1}{2}P_{L}\left(1+\frac{Z_{0}}{Z_{L}}\right)\\ P_{\_}=\frac{1}{2}P_{L}\left(1-\frac{Z_{0}}{Z_{L}}\right) \end{array}\right)$$

Both the *P*_+_ and *P*_−_ are complex numbers as functions of frequency *f*, and they could be expressed in forms of amplitude and phase:

(5) $$\left\{\begin{array}{c} P_{+}=A_{+}\left(f\right)∠\theta_{+}(f)\\ P_{\_}=A_{\_}\left(f\right)∠\theta_{-}(f) \end{array}\right)$$

**Figure 3:**
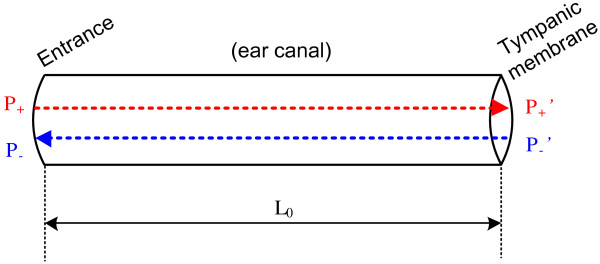
**Forward and backward sound pressures.** Forward and backward sound pressures at the entrance of the ear canal and the TM position.

Since the energy absorption by the walls of the ear canal is negligible, the forward and backward sound pressures (*P*_+_′ and *P*_−_′) at the TM could be obtained by shifting the phases of *P*_+_ and *P*_−_ by Δ*θ*(*f*) for traveling a distance of the effective length (*L*_0_) along the ear canal, while keeping the amplitudes unchanged (Figure 3):

(6) $$\left\{\begin{array}{c} {P_{+}}^{'}=A_{+}\left(f\right)∠\left[\theta_{+}\left(f\right)+\Delta \theta \left(f\right)\right]\\ {P_{-}}^{'}=A_{-}\left(f\right)∠\left[\theta_{-}\left(f\right)+\Delta \theta \left(f\right)\right] \end{array}\right)$$

The effective length *L*_0_ could be calculated by the first notch frequency ( *f*_0_) of the *P*_*L*_ spectrum by *L*_0_ = *c*/4*f*_0_ according to the quarter wavelength theory. Meanwhile, *L*_0_ was also related with the phase shift Δ*θ*( *f*) by *L*_0_ = *c*Δ*θ*( *f*)/2*πf* according to basic acoustics. By substituting the *L*_0_ in the two equations, the phase shift Δ*θ*( *f*) could be calculated by:

(7) $$\Delta \theta \left(f\right)=\frac{\mathrm{\pi f}}{2f_{0}}$$

With the Δ*θ*(*f*)calculated for all frequencies, the *P*_+_′ and *P*_−_′ could be determined by Equation (6). Finally, the estimated TM sound pressure ${\hat{P}}_{\mathrm{TM}}$ was obtained by the vector summation of *P*_+_′ and *P*_−_′:

(8) $${\hat{P}}_{\mathrm{TM}}={P_{+}}^{'}+{P_{-}}^{'}$$

**Figure 4:**
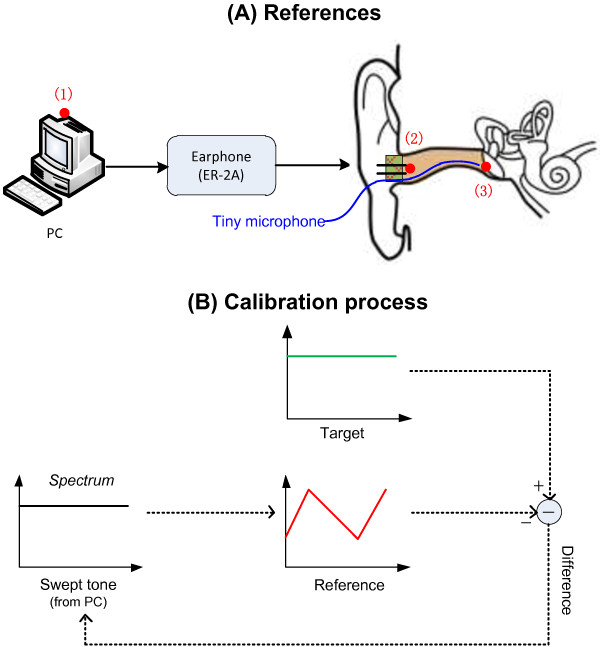
**Calibration process. (A)** Different reference SPLs for different calibration methods: 1) no calibration; 2) probe calibration; 3) in-situ calibration; **(B)** The flowchart for the calibration process.

To validate the accuracy of the estimated ${\hat{P}}_{\mathrm{TM}}$, a tiny microphone was inserted deep into the ear canal within 2 mm to the TM (Figure 4). An otoscope was used to monitor the insertion process to avoid any possible damage to the TM. Then the actual TM sound pressure *P*_*TM*_ was measured by the tiny microphone and the results were compared with the estimated ${\hat{P}}_{\mathrm{TM}}$ in the frequency domain.

### Effects of in-situ calibration on swept-tone SFOAEs

The major objective of this study was to propose an in-situ calibration that was based on the estimated TM SPL [calculated from Equation (1)-(8)] to improve the accuracy of the stimulus calibration in OAE measurements. To examine the benefits of the proposed in-situ calibration, two other calibration methods currently used in the clinic (the “no calibration” and “probe calibration”) were also involved as a comparison. The calibration process was the same for all the three different calibration methods [Figure 4(B)], except that different SPLs were used as the reference during the calibration.

For the “in-situ calibration”, the estimated TM SPL ${\hat{P}}_{\mathrm{TM}}$ at position (3) in Figure 4(A) was used as the reference SPL. Initially, a swept-tone stimulus with 0.2 volt across frequencies was generated from the PC to play the sound and the ${\hat{P}}_{\mathrm{TM}}$ was estimated using Equation (1)-(8). Then the amplitude difference between the estimated ${\hat{P}}_{\mathrm{TM}}$ and a preset target (50 dB SPL across frequencies from 0.5 to 10 kHz) was calculated at all frequencies. Then the amplitude difference was used to digitally modify the swept tone in the frequency domain from the PC. As a result, the new estimated ${\hat{P}}_{\mathrm{TM}}$in response to the modified swept-tone would be more similar to the target. The calculation process in Figure 4(B) continued until the maximal difference between the estimated ${\hat{P}}_{\mathrm{TM}}$ and the target was less than 0.1 dB across frequencies. The calibration process of the “probe calibration” method was similar, with the only exception that the reference SPL was changed to the sound pressure at the ear-canal entrance (*P*_*L*_) at position (2) in Figure 4(A). In contrast, the “no calibration” method did not take into account the standing-wave problem and the voltage of the swept tone was kept the same and globally changed for all frequencies until the measured *P*_*L*_ was 50 dB SPL at 1 kHz.

About 10 min later when the subjects (3 male and 3 female) finished the experiment of TM SPL estimate, they participated in this part of the experiment. During this part of experiment, each subject was quietly seated in the sound-proof booth with the foam probe was inserted to the left ear, and SFOAEs evoked by swept tones with time-varying frequencies were measured according to the experimental procedures described by Chen et al. [24] under three different calibration conditions (“no calibration”, “probe calibration” and “in-situ calibration”), successively. A dynamic tracking filter [24] was used to separate the swept-tone SFOAEs from various interfering noises. Then the amplitude spectra of the swept-tone SFOAEs were compared among different calibrations to examine their effects on OAE measurements.

## Results

### Estimate of sound pressure level at TM

In this study, the SPL at the TM could be indirectly calculated by the SPL measured at the entrance of the ear canal and the acoustic characteristics of the earphone system. Therefore, the accuracy of the earphone characteristics (*P*_*s*_ and *Z*_*s*_), obtained by measuring acoustic responses of a set of tubes, played an important role in the estimate of the TM SPL. In the experiment, a total of 30 different lengths of tubes were measured and the responses were evenly divided into 6 groups, with each group consist of 5 equations to solve for *P*_*s*_ and *Z*_*s*_. Then the spectra of the 6 solutions were systemically compared to examine the reliability and the average of the solutions was used to improve the accuracy of *P*_*s*_ and *Z*_*s*_.

**Figure 5:**
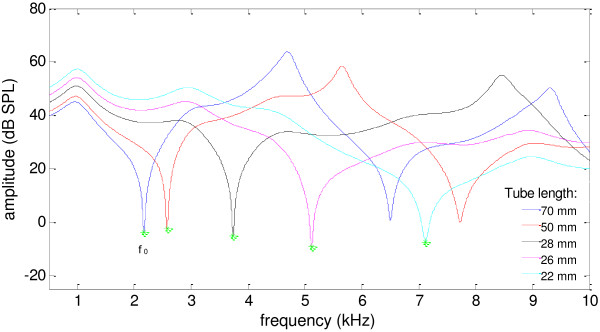
**Sound pressure levels at the tube entrance.** The amplitude spectra of the sound pressure levels at the entrance of the tube (*P*_*i*_) as functions of five effective tube length. The asterisks indicate the first notch frequency (*f*_0_) of *P*_*i*_.

**Figure 6:**
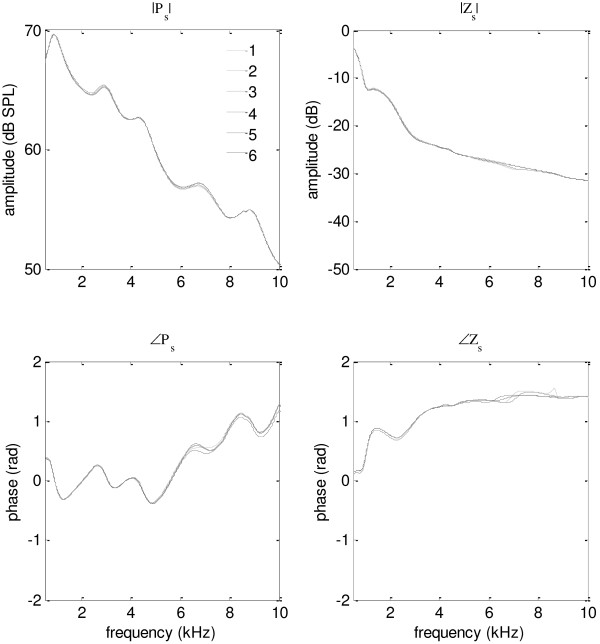
**Source pressure and source impedance.** The amplitude and phase of the source pressure (*P*_s_) and source impedance (*Z*_s_) of the earphones from six different solutions.

A typical example of the acoustic responses from 5 tube lengths that were used to solve *P*_*s*_ and *Z*_*s*_ was shown in Figure 5. It was observed that there were apparent peaks and notches for the sound pressures (*P*_i_) measured at the entrance of the tube, due to the impacts of the standing wave. The first notch frequency *f*_0_ was a decreasing function of the effective tube length. The notch frequencies were measured for each *P*_i_ to obtain a more accurate quantification of the effective tube lengths (according to the quarter wavelength theory) to further improve the accuracy of *P*_*s*_ and *Z*_*s*_, according to Equation (1)-(2). Then the solutions of the earphone parameters obtained from the six equation groups were compared in Figure 6. It was observed that the results of the 6 different solutions demonstrated great consistency and reliability, indicated by the closely overlapped curves for both the amplitude and phase. The maximal differences between solutions were 1.12 dB and 0.90 dB (both at 6.8 kHz) for the amplitude of *P*_*s*_ and *Z*_*s*_, respectively, while the maximal phase differences were 0.20 rad (at about 7 kHz) and 0.17 rad (at 8.7 kHz) for *P*_*s*_ and *Z*_*s*_. The deviations among solutions seemed to be smaller for lower frequencies for both the amplitude and phase curves.

**Figure 7:**
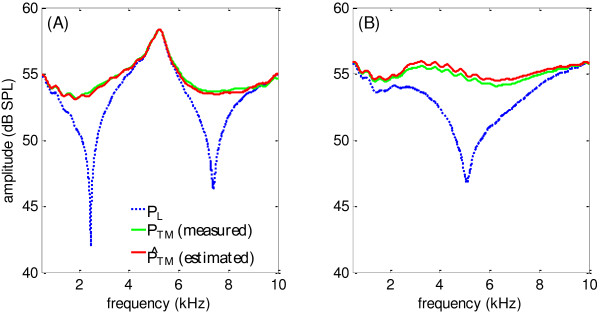
**Measured and estimated sound pressures.** The SPL at the ear-canal entrance (*P*_*L*_), the esitimated SPL (${\hat{P}}_{\mathrm{TM}}$) and measured SPL (*P*_*TM*_) at the TM from two different subjects. **(A)**: subject # 5; **(B)** subject # 3.

Since direct measurements of TM sound pressure are of great difficulties in clinical practices, it is necessary to estimate it so that deep insertions of a microphone into the ear canal could be avoided. In this study, the TM sound pressure (${\hat{P}}_{\mathrm{TM}}$) was estimated by measuring the SPL at the entrance of the ear canal (*P*_*L*_), and the validity was examined by the comparison with the actual SPL (*P*_*TM*_) measured near the TM. Two typical examples (subject # 5 and # 3) of the validations were shown in Figure 7. It was observed from Figure 7(A) that the SPLs at different positions of the ear canal demonstrated prominent differences because of the standing wave: while the SPL at the ear-canal entrance *P*_*L*_ showed deep notches around 2.5 and 7.5 kHz, the SPL at the TM position demonstrated neither of these notches. Meanwhile, a peak was present around 5.2 kHz for the SPLs at both the entrance and the TM position. In Figure 7(B), large differences between them were also observed around the notch frequency although *P*_*L*_ and *P*_*TM*_ demonstrated quite different patterns. Another important finding was that the estimated ${\hat{P}}_{\mathrm{TM}}$ matched the measured *P*_*TM*_ well across frequencies from 0.5 to 10 kHz for both subjects, suggested by the closely overlapped amplitude curves of the two sound pressures. The maximal difference between the estimated and measured TM SPL was 1.3 dB for subject # 5 and 1.6 dB for subject # 3. The estimate error seemed to slightly increase around the notch frequencies for both subjects. The estimates of the TM SPL were also quite valid for the other four subjects participated in this experiment, with a maximal estimate error of 3.2 dB across subjects. The patterns of *P*_*L*_ and *P*_*TM*_ varied for different subjects, depending on the insertion depth of the foam probe and physical properties (mainly the length and shape) of the ear canal.

### Effects of in-situ calibration on swept-tone SFOAEs

**Figure 8:**
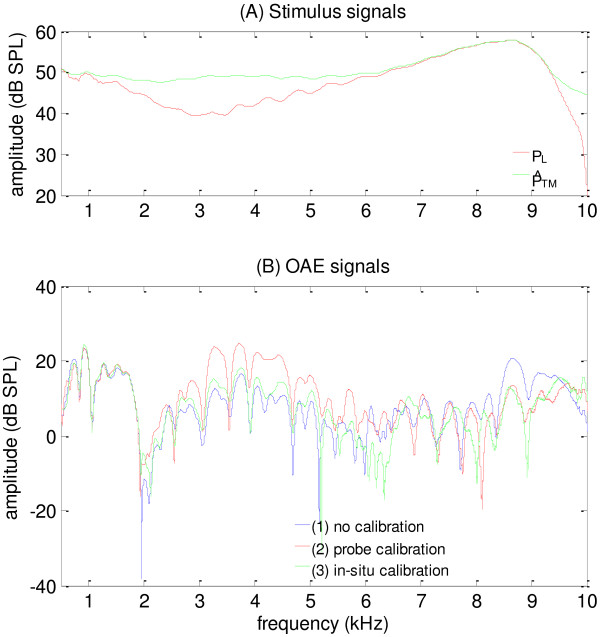
**Effects on swept-tone SFOAEs.** The effects of different calibration methods on swept-tone SFOAEs. **(A)** Stimulus signals at the entrance and the TM of the ear canal; **(B)** Amplitude spectra of OAE signals from different calibration methods.

In this study, an in-situ calibration based on the estimated TM SPL was proposed in the measurement of SFOAEs evoked by swept tones, and the results were compared with two other calibration methods (“no calibration” and “probe calibration”) currently used in the clinic. A typical example (subject # 2) of the effects of different calibrations on swept-tone SFOAEs were shown in Figure 8. The SPL measured at the entrance of the ear canal (*P*_*L*_) and the estimated TM SPL (${\hat{P}}_{\mathrm{TM}}$) were represented in Figure 8(A), and the swept-tone SFOAEs measured under three different calibration conditions were compared in Figure 8(B). It could be observed from Figure 8(B) that all swept-tone SFOAEs exhibited similar patterns as the findings from other studies [24,28,29]: a global baseline fluctuation superimposed with periodic fine structures (indicated by local peaks and notches). However, evident differences were observed among different calibrations. For the no calibration method, the sound energy entering the middle ear [represented by ${\hat{P}}_{\mathrm{TM}}$ in Figure 8(A)] demonstrated a peak of about 10 dB higher than surrounding frequencies at around 8.5 kHz. As a consequence, the SFOAEs at the corresponding frequency showed an evident elevation, which was not observed for the other two calibrations. For the probe calibration, extra gains were applied to the swept tone around 3.5 kHz (the notch frequency of *P*_*L*_) to equalize *P*_*L*_ across frequencies. As a result, there was an over compensation around the notch frequency for the sound delivered to the middle ear and the swept-tone SFOAEs showed an elevation of over 7 dB around 3.5 kHz, compared with the SFOAEs of the other two calibrations. In contrast, the sound energy delivered to the middle ear was nearly constant at different frequencies (by equalizing ${\hat{P}}_{\mathrm{TM}}$) for the in-situ calibration, and the corresponding SFOAEs did not show any apparent elevations because of larger stimulus level at the standing-wave frequencies. The SFOAEs from the in-situ calibration were most correlated with the hearing thresholds obtained from the audiogram tests prior to the experiment.

## Discussion

This study demonstrated that the presence of standing waves caused problems in the quantifications of the sound pressures within the ear canal. It is necessary to calibrate the stimulus level so that the exact amount of sound energy entering the middle ear could be precisely controlled. Since the direct measurement of the TM SPL, a common reference of the sound entering the ear, causes pains and is rarely used in the clinic, this study proposed an alternative method to estimate the TM SPL.

### The standing wave problem

It is demonstrated by this study that the standing wave has large impacts on the SPL measurements in the ear canal. Although the swept tone digitally generated from the PC has a constant driving voltage of 0.2 V from 0.5 to 10 kHz, the actual SPL *P*_*L*_ measured at the ear-canal entrance shows a completely different spectral shape (Figure 7). Given that the frequency response of the ER-2A earphone is rather flat within our measured frequency range, the shape of *P*_*L*_ should be caused by the presence of the standing wave. The forward and backward waves are out of phase at 2.5 and 7.5 kHz in Figure 7(A), and they could cancel each other to form a pressure notch at these frequencies. The frequencies and depths of the notches varied from subject to subject, mainly depending on the geometry of the ear canal, the insertion depth of the foam probe and the reflectance of the TM [2,4,30].

On the other hand, pressure peaks could also be observed in the sound pressure *P*_*L*_ when the forward and backward waves are in phase (Figures 7 and 8). The peaks are not discussed as much as the notches in relevant studies exploring the standing-wave effects [1,15,30,31]. However, the amplitude of the peaks could be over 10 dB above the level of surrounding frequencies, and therefore did cause observable elevations of the SFOAE amplitudes at the corresponding frequency (Figure 8). The peaks could possibly cause similar problems in other hearing measurements if not well taken care of, such as discomforts of patients in hearing aid fittings.

Another importance finding about the standing-wave problem is that the SPLs measured at the ear-canal entrance and TM position demonstrated large differences, as indicated by the comparison between *P*_*L*_ and *P*_*TM*_ (or ${\hat{P}}_{\mathrm{TM}}$) in Figure 7. The most significant difference is that deep pressure notches could be observed when SPL was measured at the entrance of the ear canal, but not at the TM position. The reason is that the forward and backward waves are no longer out of phase at the notch frequency after they travel from the ear-canal entrance to the TM position. The difference between *P*_*L*_ and *P*_*TM*_, especially around the notch frequency of *P*_*L*_, should be taken into consideration in stimulus level calibrations, otherwise the stimulus could be largely over-compensated [1,11].

### Estimate of sound pressure level at TM

One original contribution of this study is that it proposed a method that is capable of estimating the sound pressure at the TM position (*P*_*TM*_) to solve the standing-wave problem. With this method, the sound energy entering the middle ear could be controlled more precisely compared with other studies. The method is non-invasive and painless since only the SPL at the ear-canal entrance needs to be measured. Unlike the forward pressure [12,14] that cannot be measured anywhere in the ear canal, the estimate of *P*_*TM*_makes a further improvement to synthesize the forward and backward waves at the TM position [Equation (8)], so that the validity of the estimated sound pressure can be actually verified. Our results showed that the proposed method could be used to estimate the TM SPL reliably, indicated by the close match between ${\hat{P}}_{\mathrm{TM}}$and *P*_*TM*_ in Figure 7. The maximal estimate error across all the six subjects participated in this study was 3.2 dB, which was within the acceptable deviations during the stimulus calibrations [32-34]. Therefore, the proposed method of estimating *P*_*TM*_ could be a useful tool for accurate controls of the SPL near the TM position in auditory research and clinical tests.

The most important procedure in eatimating *P*_*TM*_ is the solution of the acoustic parameters of the earphone (*P*_*s*_ and *Z*_*s*_). Although two equations are enough to solve two unkown variables, the accuracy of the solution will decline at the frequencies where either sound pressure *P*_*i*_ [Equation (1)] shows a notch, since the SPL measurement at the notches is more affected by random noises. Therefore, five or more euqations are recommended by the investigators for more accurate solution of *P*_*s*_ and *Z*_*s*_ using a least square error method [12,14,26]. The use of the calculation tube with movable piston make it more convenient than fixed tubes to provide as many equations as needed. For the choice of piston positions, it was found in this study that the first notch frequencies of the *P*_*i*_’s should be evenly distributed within the measured frequency range (0.5-10 kHz) for optimal solutions. The comparisons between multiple solutions of *P*_*s*_ and *Z*_*s*_ calculated from different sets of *P*_*i*_ and *Z*_*i*_ suggest that the transmission line model is rather reliable when used to obtain the earphone parameters. The ripples observed in the spectra of *P*_*s*_ and *Z*_*s*_ were mainly introdcued by the resonance of the tube that connected the ER-2A earphone and the eartip in the Etymotic earphone system.

### Effects of calibration methods on SFOAEs

Another contribution of this paper is the use of swept tones in OAE measurements. The swept-tone technology makes it possible to capture any slight difference in OAE amplitude caused by difference in the stimulus level and obtain a full picture of the calibration effects on OAE measurements. OAEs of different frequencies are commonly compared to identify the frequency range with possible hearing loss [35-37], under the condition that they are evoked by stimuli of the same level. Therefore, the desired spectrum is usually kept flat across frequencies in OAE measurements. Given that the SPL at the TM position is preferably used to reflect the sound energy entering the middle ear, a good calibration method should adjust the swept tone to achieve a desired flat spectrum at the TM position, which is an important factor to consider when examining the effects of different calibrations on SFOAE measurements.

An important finding of this study is that different calibrations have significant effects on the amplitudes of the swept-tone SFOAEs (Figure 8). The effects could be explained by the difference in the TM SPL (*P*_*TM*_) of different calibration methods, since the OAE amplitude is closely related with the level of the evoking stimulus in common practices. For the no calibration method, the stimulus level of *P*_*TM*_ has a peak around 8.5 kHz due to the presence of standing waves, and the SFOAE showed a significant increase at the corresponding frequency. For the probe calibration, it falsely equated *P*_*L*_ with *P*_*TM*_, regardless of the fact that the pressure notch in *P*_*L*_ does not exist in *P*_*TM*_. A gain as much as 10 dB was added around 3.5 kHz to achieve a flat *P*_*L*_ and therefore *P*_*TM*_ was over compensated by nearly the same amount, causing significant OAE elevation around the corresponding frequency (Figure 8). For both the no calibration and probe calibration, the significantly higher amplitude of SFOAEs does not reflect better hearing ability, but is instead caused by the undesired higher stimulus level. In contrast, the in-situ calibration is not confounded by the standing wave. It ensures that the SPL at the TM has constant amplitude across frequencies and therefore the corresponding results could be more truthful to reflect the functional status of the inner ear.

There are arguments on whether different calibration methods could lead to observable changes of OAEs. For example, Siegel [11], Scheperle et al. [12] and Burke et al. [9] reported that the level of distortion product otoacoustic emissions (DPOAEs) exhibit significant discrepancy among different calibration methods, especially at high frequencies above 5–7 kHz. The calibration method based on the forward pressure level or the TM sound pressure yields less variable test results and is recommended by these studies. However, Rogers et al. [15] found little difference in either the DPOAE levels or the hearing thresholds between different calibrations, and they did not suggest a need to change the current calibration methods (no calibration or probe calibration). One possible explanation for the contradictions between these studies is that DPOAEs were performed at discrete frequencies with insufficient resolutions. As noted in Figure 8, SFOAEs showed maximal differences around the notch or peak frequencies of the standing wave and they were rather similar at other frequencies. The discrete frequencies where DPOAEs were measured by Rogers et al. [15] were 2, 3, 4, 6, and 8 kHz, and they may not be around the standing-wave notches or peaks for their participants, leading to observations of insignificant DPOAE differences among calibrations. In this study, a much larger number of frequencies could be measured using the swept-tone technique and OAE results of very high definitions could be used to capture differences at any frequencies introduced by different calibrations. On the other hand, the generation mechanisms of SFOAEs are simpler than DPOAEs [19,38], making SFOAEs more suitable to investigate the effects of calibrations on OAE measurements. In future studies, the in-situ calibration and swept-tone technology could also be used in the measurements of DPOAEs to obtain high-resolution results without the contamination of standing waves. The results of this study suggest that the proposed in-situ calibration might be incorporated into the protocols of clinical OAE measurements to improve the accuracy of the results.

## Conclusions

In this study, it was demonstrated that the standing wave in the ear canal has large impacts on the quantification of sound energy entering the middle ear. Therefore, a transmission line model was used to estimate the SPL at the TM position to precisely control the acoustic energy delivered to the middle ear. Then an in-situ calibration based on the estimated TM SPL was proposed to control the primary level of SFOAEs evoked by swept tones. The comparison of the high-definition SFOAE spectra among different calibrations demonstrated that the methods currently used in the clinic may produce inaccurate results around standing-wave frequencies. In contrast, the in-situ calibration was not affected by the standing wave and might be a great candidate for more accurate diagnoses of the functional status of the inner ear in audiologic measurements.

## Abbreviations

TM: Tympanic membrane; SPL: Sound pressure level; SFOAEs: Stimulus frequency otoacoustic emissions; DPOAEs: Distortion product otoacoustic emissions.

## Competing interests

The authors of this paper report that they have no biomedical financial interests or potential conflicts of interest.

## Authors’ contributions

SC and GL designed the experimental protocols. SC and HZ carried out the experiment and collected the data. LW was responsible for the data analyses. All authors were involved in writing and revising the manuscript. All authors read and approved the final manuscript.
